# Piperlongumine selectively kills cancer cells and increases cisplatin antitumor activity in head and neck cancer

**DOI:** 10.18632/oncotarget.2402

**Published:** 2014-08-27

**Authors:** Jong-Lyel Roh, Eun Hye Kim, Jin Young Park, Ji Won Kim, Minsu Kwon, Byung-Heon Lee

**Affiliations:** ^1^ Department of Otolaryngology, Asan Medical Center, University of Ulsan College of Medicine, Seoul, Republic of Korea; ^2^ Department of Biochemistry and Cell Biology, School of Medicine, Kyungpook National University, Daegu, Republic of Korea

**Keywords:** piperlongumine, reactive oxygen species, head and neck cancer, cisplatin, cell death

## Abstract

Adaptation to cellular stress is not a vital function of normal cells but is required of cancer cells, and as such might be a sensible target in cancer therapy. Piperlongumine is a naturally occurring small molecule selectively toxic to cancer cells. This study assesses the cytotoxicity of piperlongumine and its combination with cisplatin in head-and-neck cancer (HNC) cells *in vitro* and *in vivo*. The effect of piperlongumine, alone and in combination with cisplatin, was assessed in human HNC cells and normal cells by measuring growth, death, cell cycle progression, reactive oxygen species (ROS) production, and protein expression, and in tumor xenograft mouse models.

Piperlongumine killed HNC cells regardless of p53 mutational status but spared normal cells. It increased ROS accumulation in HNC cells, an effect that can be blocked by the antioxidant *N*-acetyl-L-cysteine. Piperlongumine induced selective cell death in HNC cells by targeting the stress response to ROS, leading to the induction of death pathways involving JNK and PARP. Piperlongumine increased cisplatin-induced cytotoxicity in HNC cells in a synergistic manner *in vitro* and *in vivo*. Piperlongumine might be a promising small molecule with which to selectively kill HNC cells and increase cisplatin antitumor activity by targeting the oxidative stress response.

## INTRODUCTION

Head and neck cancer (HNC) is the eighth most common cancer worldwide, with more than half a million new cases diagnosed each year [1]. The overall incidence of HNC in the United States is declining despite a rising trend of oropharyngeal cancer incidence associated with oncogenic human papilloma virus (HPV) [2, 3]. Tobacco and alcohol consumption increase the risk of developing HNC, which is largely attributable to the genotoxic effects of the carcinogens in these substances [4]. Over 50% of HNC display chromosomal loss at 17p, the site of the *TP53* gene, or harbor inactivating *TP53* gene mutations, particularly in HPV-negative HNC [5, 6]. HNC with *TP53* mutations is generally less responsive to chemoradiation and shows poorer survival than HPV-positive HNC, which commonly does not harbor *TP53* mutations [5, 7].

Current treatment modalities for HNC include surgery, radiotherapy, chemotherapy, and their combinations [8]. Despites recent advances in the diagnosis and treatment of HNC, overall survival has not substantially changed over the last three decades [8]. This may result from the fact that alterations in tumor suppressor genes or signaling pathways are associated with therapeutic resistance [9]. Gain-of-function mutations in oncogenes and loss-of-function mutations in tumor suppressor genes result in increased cellular stress not ordinarily observed in normal cells [10]. Targeting cancer-specific deregulation, such as oxidative or metabolic changes, may result in the selective death of cancer cells [11, 12].

Piperlongumine (PL), a natural product isolated from the long pepper *Piper longum* L. [13], was recently identified as selectively toxic to cancer cells *in vitro* and *in vivo* [14]. PL was identified in a cell-based high-throughput screen designed to find compounds with novel pro-apoptotic mechanisms [14]. PL elevates ROS cellular levels and selectively induces apoptotic death in cancer cells, with no apparent toxicity in normal cells [14, 15]. Although tested in several types of human malignancies [16-20], PL has not yet been tested in HNC. Further investigation of its ROS-dependent and -independent mechanisms and of its synergy with conventional chemotherapeutic agents is needed [15]. Here, we show that PL selectively kills HNC cells by targeting the oxidative stress response and increases the antitumor activity of cisplatin, a first-line chemotherapeutic agent used in HNC therapy.

## RESULTS

### Piperlongumine selectively kills HNC cells but not normal cells

**Figure 1:**
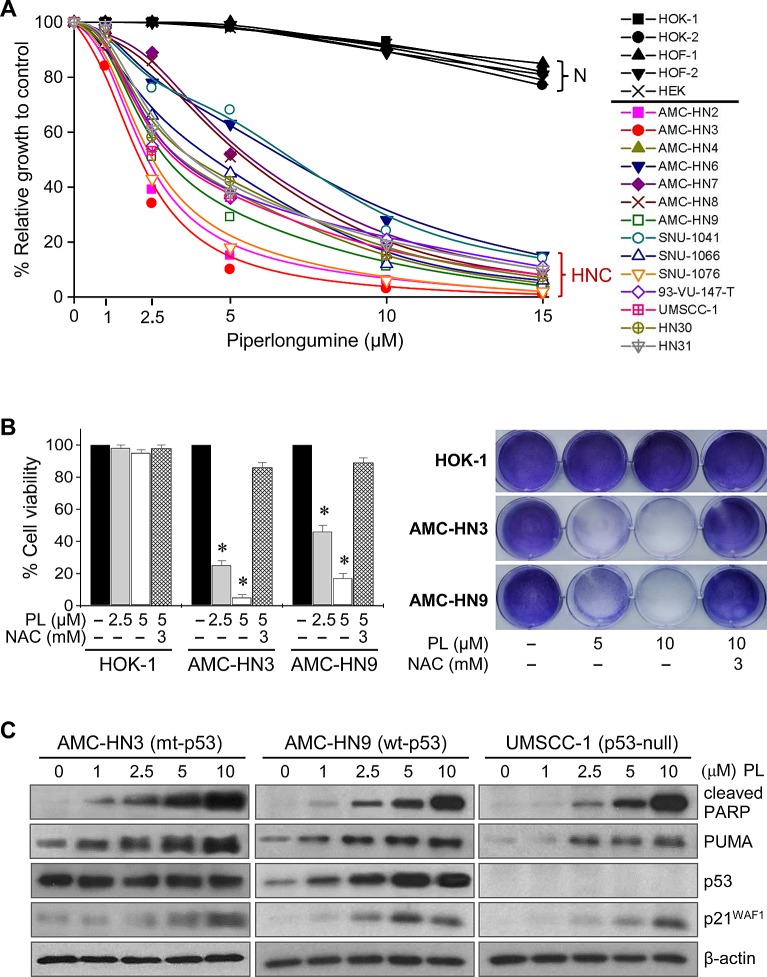
Piperlongumine selectively kills HNC cells (A-B) Piperlongumine induces death in HNC cells but not normal cells. Cytotoxicity was assessed by MTT assay (A), trypan blue exclusion assay, and crystal violet staining (B) after exposure to 1–15 μM piperlongumine (PL) for 48–72 h. Normal human cells (N) included oral keratinocytes (HOK), oral fibroblasts (HOF), and skin keratinocytes (HEK) isolated from human oral mucosa and skin, respectively. The cytotoxic effect of PL was blocked by the antioxidant *N*-acetyl-L-cystine (NAC, 3 mM). The error bars represent s.d. from three independent experiments, each performed with triplicate samples. * denotes *p* < 0.001 relative to control. (C) Western blot analysis revealing changes in levels of p53 and its targets, cleaved PARP, PUMA, and p21^WAF1^, in several HNC cells with mutant (mt), wide-type (wt), or null p53 exposed to PL for 24 h. β-actin level was assessed as a loading control.

The cytotoxic effects of PL were tested in cultured human HNC cells and normal cells. PL markedly induced death in cancer cells, while the viability of normal cells was affected only minimally at the highest concentration (15 μM) tested (Figure 1). The cytotoxicity of PL was blocked by pretreatment with the antioxidant NAC, indicating that PL might selectively kill cancer cells, including HNC cells, in which an active response to oxidative stress occurs. Western blot analysis showed that PL significantly increased the expression of wild-type p53, of the p53 proapoptotic targets PARP and PUMA, and of p21 in AMC-HN9 cells. PL also increased the levels of proapoptotic proteins in mutant p53 (R282W)-expressing AMC-HN3 cells and in p53-null UMSCC-1 cancer cells. This suggests that PL selectively induces cancer cell death by modulating the expression of apoptotic and survival pathways regardless of p53 status.

### Piperlongumine selectively increases ROS accumulation in HNC cells

**Figure 2:**
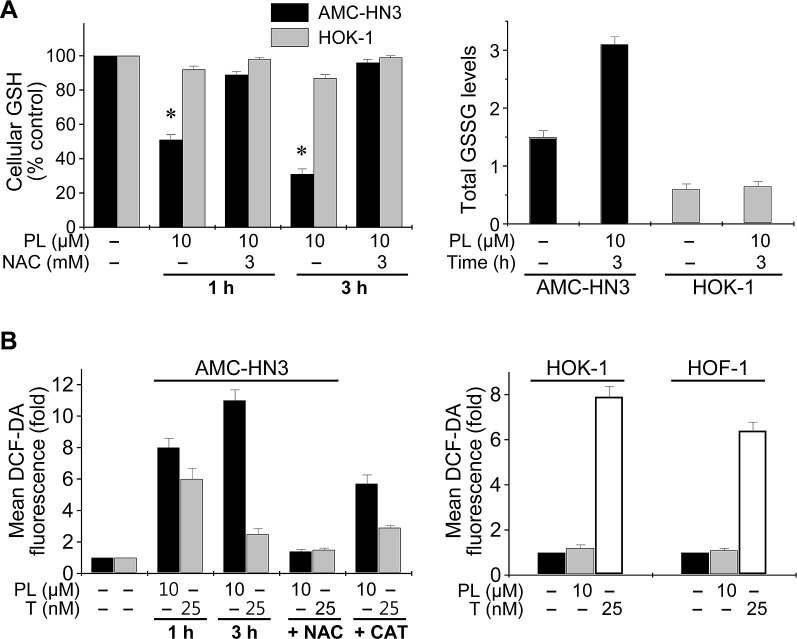
Piperlongumine selectively increases ROS accumulation in HNC cells but not normal cells (A) Modulation of cellular GSH and GSSG levels by piperlongumine (PL). The GSH and GSSG levels were measured after AMC-HN3 and HOK-1 cells were exposed to PL for 1 h and 3 h with or without 3 mM NAC pretreatment for 1 h. The error bars represent s.d. from three independent experiments, each performed with triplicate samples. * denotes *p* < 0.001 relative to control. (B) ROS elevation by PL and prevention of the effect by NAC or catalase. AMC-HN3 or normal cells (HOK-1 and HOF-1) were exposed to 10 μM PL, paclitaxel (T, 25 nM) or DMSO (basal) for 1 h and 3 h. Cells were also pretreated with NAC (3 mM) for 1 h or catalase (CAT, 2,000 UmL^−1^) for 2 h before exposure to PL (10 μM) or paclitaxel (25 nM) for 3 h. ROS levels were measured by flow cytometry using DCF-DA and are shown as fold changes over DMSO-treated (basal) levels. Histograms are representative of three separate experiments. PL increased ROS levels in HNC cells (left panel), but not in normal cells (right panel). All values are the mean ± s.d. of three independent experiments.

PL targets proteins regulating oxidative stress [14]. When the glutathione (GSH) and glutathione disulfide (GSSG) levels were measured after HNC cells and normal HOK-1 cells were exposed to PL for 1 h and 3 h, results showed that PL decreased GSH levels and increased GSSG levels in HNC cells (Figure 2 and Supplementary Figure S1); however, PL did not increase GSSG levels in normal HOK-1 cells. Further, the reducing agent NAC, which extinguishes cellular ROS, prevented PL-mediated GSH depletion. Next, the effect of PL on cellular ROS levels in HNC and HOK-1 cells was assessed by flow cytometry using the redox-sensitive fluorescent probe DCF-DA. Exposure to PL for 1 h and 3 h caused a significant increase in ROS levels in HNC cells but not in normal HOK-1 cells. Exposure to paclitaxel for 1 h also increased ROS levels in HNC cells; however, that effect was reduced after 3 h, which is in contrast to the sustained elevation of cellular ROS levels observed upon exposure to PL. In addition to cancer cells, paclitaxel induced a marked increase in DCF-DA fluorescence in normal HOK-1 and HOF-1 cells, which PL did not do. Co-exposure with NAC or catalase blocked the PL-induced ROS increase in cancer cells.

### Piperlongumine induces cell cycle changes and cell death

**Figure 3:**
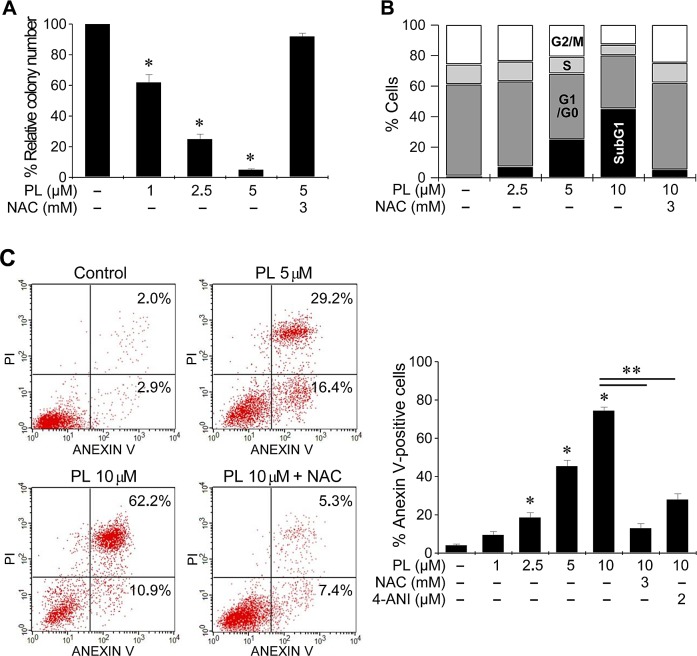
Piperlongumine induces cell cycle changes and cell death (A) Clonogenic assay of cancer cell lines exposed to PL. AMC-HN3 cancer cells were exposed to PL or DMSO (control) for 48 h. The error bars represent the s.d. from three independent experiments, each performed in triplicate. * denotes *p* < 0.01 relative to control. (B) Cell cycle analysis after exposure to PL. AMC-HN3 cells exposed to DMSO or PL for 48 h were stained with propidium iodide and subjected to flow cytometry analysis. (C) Apoptosis assays in AMC-HN3 cells exposed to PL. Cells were exposed to PL for 48 h, and the annexin V-positive apoptotic fractions were measured. *, ** denote *p* < 0.05 relative to control and 10 μM PL, respectively. Cells were also pretreated with 3 mM NAC for 1 h or 2 μM of the PARP inhibitor 4-amino-1,8-naphthalimide (4-ANI) for 16 h before being exposed to PL (5 or 10 μM).

PL induced a marked decrease in the number of cancer cell colonies (Figure 3 and Supplementary Figure S2). In cell cycle analysis by flow cytometry using propidium iodide staining in AMC-HN3 cells, PL increased the sub-G1 apoptotic population, and that effect was blocked by co-exposure to NAC. Further, apoptosis assays showed that PL induced a significant increase in apoptosis and cell death in HNC cells. Co-exposure to PL and the antioxidant NAC or the PARP inhibitor 4-ANI protected cancer cells from apoptosis.

### Piperlongumine induces cancer cell death by interfering with ROS regulators

**Figure 4:**
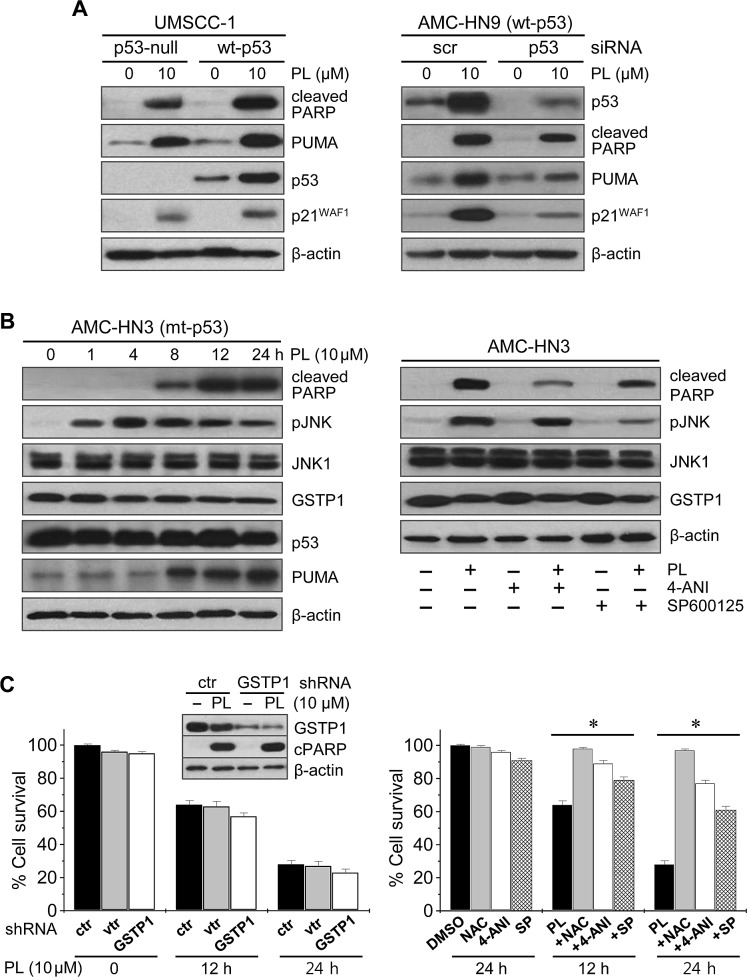
Piperlongumine induces cancer cell death by interfering with ROS regulators (A) The effects of PL on p53 and its target proteins, PUMA, cleaved PARP and p21^WAF1^, were measured by Western blot analysis in cancer cells exposed to 10 μM PL or DMSO (control). Wild-type (wt) p53 was stably transfected in p53-null UMSCC-1 using a retroviral vector. AMC-HN9 cells with wt p53 were transfected with scrambled siRNA (scr) or p53 siRNA for 48 h, prior to PL exposure. (B) Western blot analysis revealing changes in levels of cleaved PARP, phospho-JNK (pJNK), JNK1, GSTP1 and PUMA. Cell extracts were obtained after exposing mutant p53 (mt-p53) AMC-HN3 cells to 10 μM PL (left panel). Cells were also pretreated with 2 μM of the PARP inhibitor 4-ANI for 16 h or 20 μM of the JNK inhibitor SP600125 for 1 h before exposure to 10 μM PL for 12 h (right panel). (C) Effects of *GSTP1* knockdown and PARP or JNK inhibition on PL-induced changes in cell growth. AMC-HN3 cells were stably transfected with *GSTP1* shRNA or control shRNA in a lentiviral vector (vtr). The knockdown was confirmed by Western blotting using anti-GSTP1 antibody. Cell viability was measured by trypan blue exclusion in parental HN3 cells and sublines (left panel), and in AMC-HN3 cell lines exposed to 10 μM PL or to the combination of 10 μM PL and 3 mM NAC, 2 μM 4-ANI, or 20 μM SP600125 (SP) (right panel). The error bars represent the s.d. from three independent experiments, each performed with triplicate samples. * denotes *p* < 0.01.

Western blot analysis showed that PL increased the levels of PARP and PUMA proteins regardless of p53 status (Figure 4): the levels of these proapoptotic proteins increased in both p53-null UMSCC-1 cells and in UMSCC-1 cells transfected with wild-type p53, and in both AMC-HN9 cells expressing endogenous wild-type p53 and AMC-HN9 cells transfected with p53-siRNA, although the levels of PUMA, cleaved PARP and p21 increased to a greater extent in HNC cells with wild-type p53. Since PL targets glutathione *S*-transferase pi 1 (GSTP1) and links cellular ROS accumulation and sustained c-Jun N-terminal kinase (JNK) activation [14, 21], we assessed PL-induced changes in associated proteins and these interactions (Figure 4, Supplementary Figures S3 and S4). PL increased the levels of cleaved PARP, PUMA and phospho-JNK (pJNK). The activation of pJNK was observed as early as 1 h after PL treatment and preceded the increase in the proapoptic proteins. Co-exposure to PL and the PARP inhibitor 4-ANI did not affect the levels of JNK1 and pJNK, while the combination of PL and JNK inhibitor significantly decreased pJNK levels and cleaved PARP. In addition, PL-induced cell death was blocked by PARP or pJNK inhibition to some degree, as was observed upon co-exposure to PL and NAC. Taken together, PL regulates ROS by targeting GSTP1, a direct negative regulator of JNK [22, 23], and thereby increases JNK phosphorylation. Further, since JNK mediates cell death caused by ROS accumulation via sustained PARP activation [24, 25], PL-induced GSTP1 inhibition and pJNK activation resulted in part in PARP activation; however, the knockdown of *GSTP1* itself did not significantly affect PARP activation, cellular ROS levels, or survival in AMC-HN3 cancer cells.

### Piperlongumine increases the cytotoxicity of cisplatin in HNC cells *in vitro* and *in vivo*

**Figure 5:**
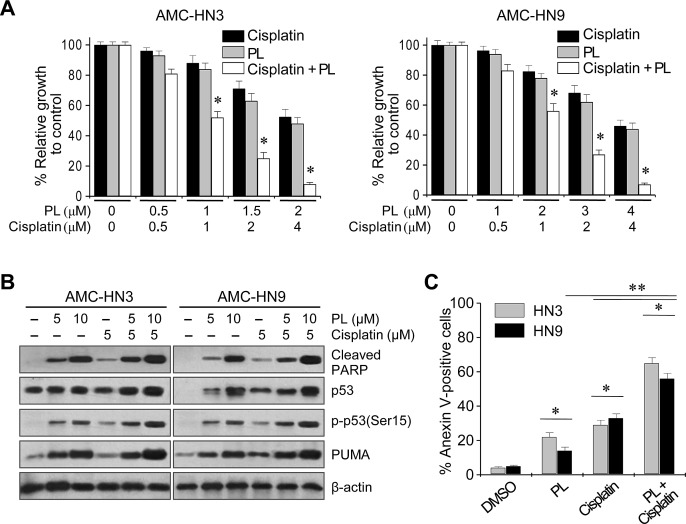
Piperlongumine increases the antitumor activity of cisplatin (A) MTT assay revealing growth inhibition by PL, cisplatin, and both drugs in combination. Cells were treated for 72 h. The error bars represent s.d. from three independent experiments, each performed with triplicate samples. * denotes combination index < 1, *p* < 0.01. (B) Western blot analysis revealed increased induction of cleaved PARP, PUMA and phospho-p53 (Ser15) proteins after combined exposure to 5 μM cisplatin and 5 or 10 μM PL for 24 h. (C) Apoptosis assays after exposure to PL, cisplatin or PL plus cisplatin. Cells were exposed to vehicle (DMSO), 2.5 μM PL, 5 μM cisplatin, or both drugs in combination for 48 h, and the annexin V-positive apoptotic fractions were measured. * denotes *p* < 0.05 in the comparison of cells exposed to PL alone, cisplatin alone, or PL plus cisplatin and control. ** denotes *p* < 0.05 in the comparison of cells exposed to PL or cisplatin alone to cells exposed to cisplatin plus PL.

We assessed the synergistic effects of PL and cisplatin. Individually, PL and cisplatin induced growth inhibition and cell death in AMC-HN3 and -HN9 cells (Figure 5). In combination, PL increased the cytotoxic activity of cisplatin in both cancer cell lines, inhibiting growth to an extent greater than the sum of the effects of either agent alone. Phospho-p53 (Ser 15), proapoptotic protein levels, and apoptosis were increased to a greater extent in AMC-HN3 and -HN9 cells exposed to the combination of PL and cisplatin than in cells exposed to each agent alone.

**Figure 6:**
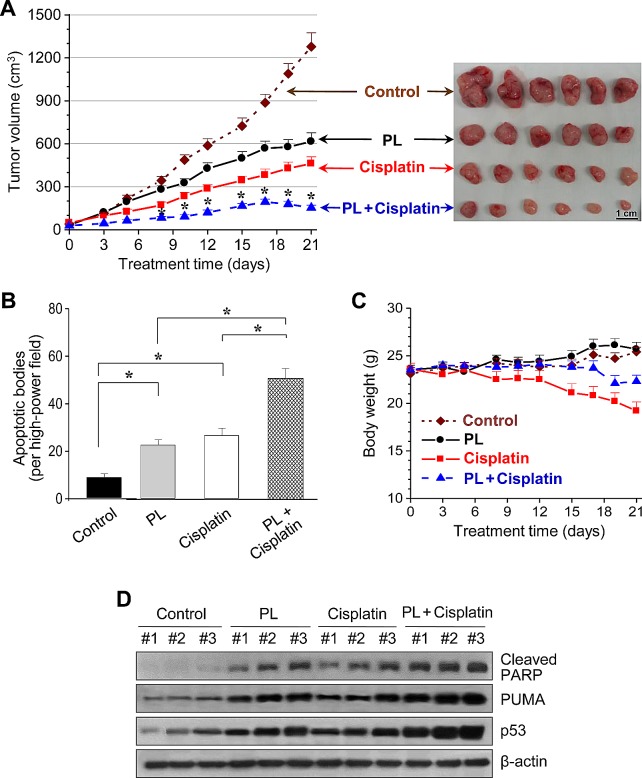
Piperlongumine and cisplatin synergistically inhibit *in vivo* tumor growth (A) Antitumor effect of PL and cisplatin in a tumor xenograft mouse model. Nude mice were injected with 5 × 10^6^ AMC-HN9 cells in both flanks. Treatments with vehicle, PL, cisplatin, or the combination of PL and cisplatin began once the implanted tumor cells formed palpable nodules. Each group included ten mice. The error bars represent standard errors. * denotes *p* < 0.05 after day 8 between groups treated with PL or cisplatin and its combination. (B) Quantification from *in situ* TUNEL assays in tumor sections from each group. TUNEL-positive apoptotic bodies were counted blindly in ten randomly selected high-powered fields. The error bars represent standard errors. Two-tailed Student's *t*-test, * denotes *p* < 0.01. (C) Changes in body weight among mice with different treatments. The error bars represent standard errors. (D) Western blot analysis of cleaved PARP, PUMA and p53 proteins obtained from tumors treated with vehicle control, PL, cisplatin, or the combination of both drugs. β-actin served as internal loading control.

We treated BALB/c athymic nude mice bearing AMC-HN3 and -HN9 tumor xenografts with i.p. injections of PL, cisplatin, PL plus cisplatin, or vehicle. PL or cisplatin alone significantly decreased the growth rate of HN3 and HN9 tumors (Figure 6 and Supplementary Figure S5). Notably, the combination of PL and cisplatin synergistically suppressed *in vivo* tumor growth. *In situ* apoptosis assays showed that TUNEL-positive apoptotic bodies were more frequently seen in tumors treated with PL-, cisplatin-, and PL plus cisplatin than in those treated with vehicle. Western blot analyses of tumor tissues showed that p53 and apoptotic protein levels were increased to a greater extent in HN9 cells treated with the combination of PL and cisplatin than in cells treated with single agents.

Changes in body weights were not significantly different between the control and PL-treated groups (*P* > 0.5). Blood analysis using an automated hematology analyzer showed that the mean values for white blood cells, lymphocytes, monocytes, hematocrits, red blood cell distribution, hemoglobin and platelet counts were not significantly different between the control and PL-treated groups (*P* > 0.2). Histopathological examination of vital organs from experimental mice did not reveal any significant difference between the control and PL-treated groups (data not shown).

## DISCUSSION

Our study shows that PL selectively killed HNC cells by perturbing redox and ROS homeostasis. PL increased ROS levels and induced selective cell death in cancer cells but not in normal cells. The targeting of a mechanism upon which the cancer cell is dependent, such as cellular ROS homeostasis, can explain the differential response of cancer and non-transformed cells to PL. In the present study, PL had the ability to inhibit the growth of rapidly growing highly aggressive HNC, regardless of p53 status. The compound caused effective cell death in HNC, even in the context of disruptive *TP53* mutations associated with aggressive disease and poor survival [5]. Therefore, targeting the stress phenotypes unique to cancer cells and their associated vulnerabilities may be a promising strategy for cancer treatment [10,11]. Our data provide the first experimental evidence supporting PL as a potential therapeutic agent for HNC.

The present study showed that PL was effective in killing HNC cells via the ROS-mediated GSTP1 and JNK pathways. Cancer cells are more dependent on elevated ROS levels and a highly functional antioxidant system than normal cells [10, 26]. The enzymatic ROS detoxification systems that prevent the induction of cell death are activated in cancer cells that resist chemotherapeutic agents [27, 28]. PL induces apoptosis by interfering with critical regulators of redox and ROS homeostasis, such as GSTP1 and carbonyl reductase 1 (CBR1) [14]. Stable overexpression of GSTP1 and CBR1 in cancer cells also reduces PL-induced ROS levels and rescues apoptosis [14]. PL targets GSTP1, which conjugates GSH to proteins during oxidative stress [15]. Since GSTP1 is a negative regulator of JNK through direct protein-protein interaction, inhibition of GSTP1 can active JNK and in turn phosphorylate c-Jun [21, 22]. Our data showed that PL activates JNK by quenching GSTP1 activity in HNC cells. Further, NAC rescued the selective PL-induced cancer cell death mediated by the ROS stress response pathway. The increased dependence of HNC cells on the oxidative stress pathway may be the basis for the selectivity of PL for cancer cells, and PL-induced cytotoxicity may be mediated by JNK signaling.

The present data suggest that PL-induced death in HNC cells might result from possible links between the phosphorylation of JNK and PARP activation. PL-induced GSTP1 inhibition and pJNK activation resulted in PARP activation. JNK mediates cell death upon ROS accumulation via sustained PARP activation [23, 24]. ROS induce cell death signaling pathways and act as cell death initiators through direct damage to various macromolecules such as proteins, DNA and lipids [29, 30]. There is evidence suggesting that JNK is an important mediator of oxidative stress-induced apoptotic and non-apoptotic/necrotic cell death in various cell types stimulated with different forms of ROS [31, 32]. In our study, high concentrations of PL induced selective non-apoptotic death in HNC cells by activation of JNK and PARP. PARP, a key DNA repair protein, was activated in cancer cells treated with PL, and pharmacological inhibition of PARP offered significant protection against PL-induced death. However, knockdown of GSTP1 did not affect PL-induced ROS levels or death in cancer cells, and pharmacological inhibition of JNK or PARP did not completely abrogate PL-induced death. These results may reflect the fact that other cell death signaling pathways may be involved. Although PL is associated with cell death in both wild-type p53 and mutant p53-harboring cancer cells, PL increases the levels of p53-Ser-15 and PUMA, which might in part explain apoptotic and non-apoptotic cell death in cancer cells [33]. In addition, recent studies showed that PL was a promising agent in several types of human malignancies targeting other pathways, e.g. p38 [17, 18] and STAT3 [20].

Our study revealed that PL synergized with cisplatin. Since cisplatin is a first-line chemotherapeutic agent used in HNC, the combination of PL and cisplatin may be effective in the clinical setting. The present study is the first to show that PL potentiates the cytotoxic effect of cisplatin in HNC cells *in vitro* and *in vivo*. PL induced a robust increase in cisplatin-mediated apoptosis via PUMA and PARP activation. HNC with loss or mutation of *TP53* is associated with cisplatin resistance through lack of senescence [34]. PL induces cell cycle arrest and inhibits angiogenesis and metastasis in cancer cells regardless of p53 status [14, 35]. PL sensitizes p53-mutant HNC cells to cisplatin, leading to increased cytotoxicity and more effective therapy for aggressive HNC. In addition, our data showed that PL did not have any apparent adverse effects *in vivo*. Taken together, these findings may be of paramount clinical significance: by inducing the death of cells with ROS accumulation, PL could reduce the dose of cisplatin required in the clinical setting and thereby minimize the potential adverse effects of cisplatin chemotherapy.

In conclusion, our data suggest that PL induces ROS accumulation and cell death selectively in HNC cells by targeting critical regulators of ROS homeostasis. The study also revealed that PL can trigger HNC cell death via JNK and PARP activation, which is a novel mechanism. Further, PL can enhance the cytotoxicity of cisplatin in both p53-wild-type and p53-mutant HNC cells. This study supports the need for further investigation of PL as a potential cancer therapy, particularly for HNC with aggressive phenotypes.

## MATERIAL AND METHODS

### Cell culture

*In vitro* assays, including cell viability, were performed in several HNC cell lines: AMC-HN2, -HN3, -HN4, -HN6, -HN7, -HN8, and -HN9 (grown in Eagle's minimum essential medium; Life Technologies^TM^, Carlsbad, CA, USA), SNU-1041, -1066, and -1076, HN30 and HN31 (grown in Roswell Park Memorial Institute medium, Life Technologies^TM^), and UMSCC1 and 93-VU-147T (grown in Dulbecco's modified Eagle medium, Life Technologies^TM^), supplemented with 10% fetal bovine serum. All cancer cell lines were authenticated by DNA (short-tandem-repeat, STR) profiling provided by the cell bank. The *in vitro* assays were also performed in normal human cells: oral keratinocytes (HOK), oral fibroblasts (HOF), and skin keratinocytes (HEK) obtained from patients undergoing surgery were grown in EpiLife^®^ serum-free cell culture medium supplemented with bovine pituitary extract (BPE) and recombinant epidermal growth factor (rEGF) (Life Technologies^TM^). The cells were incubated at 37°C in a humidified atmosphere containing 5% CO_2_.

### Cell viability assay

Cell viability was determined by trypan blue exclusion, crystal violet staining, MTT and clonogenic assays. For trypan blue exclusion, cells were seeded at 1 × 10^5^ in 6-well plates, allowed to reach 60–70% confluence, and treated with PL (Tocris Bioscience, Bristol, UK) for 48 h. The cells were then trypsinized, stained with 0.4% trypan blue (Life Technologies^TM^), and counted using a hemocytometer. For crystal violet staining, used for visual quantification of cell viability, cells were grown in 6-well plates and exposed to PL for 48 h. After the medium was removed, cells were washed in cold phosphate buffered saline (PBS), fixed with ice-cold 100% methanol, and stained with 0.5% crystal violet solution (Sigma-Aldrich, Louis, MO, USA). For MTT assays, cells were seeded at 3–5 × 10^3^ cells/well in 96-well plates, incubated overnight, and exposed to PL and cis-platinum (II) diamine dichloride (cisplatin; Sigma-Aldrich), alone or in combination, for 72 h. The cells were then exposed to the tetrazolium compound 3-[4,5-dimethyl-2-thiazolyl]-2,5-diphenyl-2H-tetrazolium bromide (MTT; Sigma-Aldrich) for 4 h, after which solubilization buffer was added for 2 h. The absorbance in each well was measured at 570 nm using a SpectraMax M2 microplate reader (Molecular Devices, Sunnyvale, CA, USA). For clonogenic assays, cells were exposed to PL or DMSO for 48 h, and then incubated in drug-free medium for 7–10 d. The wells were stained with 0.5% crystal violet solution, and the number of colonies was counted. All the assays were performed with triplicate samples and repeated three times.

The interaction of two drugs was considered synergistic when growth suppression was greater than the sum of the suppression induced by either drug alone [36]. Briefly, the combination index (CI) was calculated according to the relative fraction of cells affected: CI = 1, additive interaction; CI < 1, synergistic interaction; CI > 1, antagonistic interaction.

### Measurement of total cellular glutathione and glutathione disulfide

To assay total cellular glutathione (GSH), tumor and normal cells were exposed to PL and N-acetyl-L-cysteine (3 mM, NAC, Sigma-Aldrich). Cells (1 × 10^6^) were collected, centrifuged and lysed in 100 μL ice-cold lysis buffer for 10 min. The lysate was centrifuged for 10 min and the supernatant was used to assay GSH with a glutathione colorimetric detection kit (BioVision Inc., Milpitas, CA, USA). The total amount of GSH was measured using a fluorescence plate reader (Molecular Devices) at excitation (ex)/emission (em) = 380/460 nm. Quantification of glutathione disulfide (GSSG) was performed using a GSH/GSSG detection kit (ABcam, Cambridge, MA, USA). The drug-treated cells were collected in ice-cold buffer, homogenized and sonicated in icy water. GSH quencher (10 μL) was added and incubated for 10 min to quench GSH at room temperature. The samples were centrifuged and the supernatant was used to determine the GSSG concentration according to the manuscript protocol using a fluorescence plate reader. The change in GSSG levels in PL-treated samples compared to DMSO-treated control samples was expressed as the fold change.

### Cell cycle and cell death assays

For cell cycle assays, cells were exposed to PL for 48 h. The cells were then trypsinized, fixed overnight in ice-cold ethanol, and stained for 30 min with propidium iodide (Sigma-Aldrich) at 37^o^C. The cellular DNA content was measured using a FACScalibur flow cytometer (BD Bioscience, San Jose, CA, USA). For cell death assays, cells were cultured with PL and cisplatin, alone or in combination, or an equivalent amount of DMSO (vehicle control). After 48 h, cells were harvested, washed in ice-cold PBS, and resuspended in binding buffer. Cells were also pretreated with 3 mM NAC for 1 h or with 2 μM poly(ADP-ribose) polymerase (PARP) inhibitor 4-amino-1,8-naphthalimide (4-ANI, Sigma-Aldrich) for 16 h, before exposure to PL. Cells were then stained with annexin V-FITC (fluorescein isothiocyanate) and propidium iodide using an annexin V-FITC apoptosis detection kit (BD Biosciences, Franklin Lakes, NJ, USA). All data were analyzed using the Cell Quest software (BD Biosciences).

### Measurement of ROS production

Cells were exposed to 10 μM PL, 20 nM paclitaxel (Sigma-Aldrich), or an equivalent amount of DMSO (control) for 1 and 3 h and ROS generation was detected with 2′,7′-dichlorofluorescein diacetate (DCF-DA) (Enzo Life Sciences, Farmingdale, NY, USA). Cells were incubated with 10 μM DCF-DA for 30 min at 37^o^C, washed twice with PBS, and analyzed in a FACScalibur flow cytometer. Cells were also pretreated with 3 mM NAC for 1 h or catalase (CAT, 2,000 UmL^−1^, Sigma-Aldrich) for 2 h, before exposure to PL (10 μM) or paclitaxel (25 nM, Sigma-Aldrich) for 3 h. ROS levels were measured by flow cytometry using DCF-DA and are shown as the fold change over DMSO-treated (basal) levels.

### Immunoblotting

Tumor cells were exposed to PL or cisplatin, alone or in combination. Cells were also pretreated with 2 μM of the PARP inhibitor 4-ANI for 16 h or the c-Jun N-terminal kinase (JNK) inhibitor SP600125 (Sigma-Aldrich) for 1 h, before exposure to 10 μM PL for 24 h. Cells were lysed at 4°C in radioimmunoprecipitation assay (RIPA) buffer (Thermo Scientific, Rockford, IL, USA). Immunoblotting was performed according to standard procedures. Briefly, a total of 50 μg protein was resolved by sodium dodecyl sulfate-polyacrylamide gel electrophoresis (SDS-PAGE) on 10–12% gels, transferred to nitrocellulose polyvinylidene difluoride membranes, and probed with primary and secondary antibodies. The following primary antibodies were used: p53 (DO1) and JNK1 (Santa Cruz Biotechnology, Santa Cruz, CA, USA); and p21^WAF1/CIP1^, PUMA, cleaved poly(ADP-ribose) polymerase (PARP), phospho-p53-Ser15, phospho-JNK (pJNK), and glutathione *S*-transferase pi 1 (GSTP1) (Cell Signaling Technology, Danvers, MA, USA). β-actin (Sigma) was used as the loading control. All antibodies were diluted between 1:250 and 1:5,000.

### Transfection and infection

For knockdown of *TP53*, AMC-HN9 with wild-type (wt) p53 was seeded onto 60 mm plates in medium without antibiotics, and 18 h later was transfected with 50 nmol/L small interfering RNA (siRNA) targeting human *TP53* or a scrambled control siRNA (Santa Cruz Biotechnology). Transfections were conducted using the Lipofectamine RNAi Max reagent (Life Technologies^TM^). After 48 h, cells were exposed to PL for an additional period of 24 h and then analyzed for protein expression. For expression of p53, wt p53 was stably transfected in the p53-null UMSCC-1 cell line using a retroviral vector containing puromycine resistance (Cell Biolabs Inc., San Diego, CA, USA). For knockdown of *GSTP1*, AMC-HN3 cells were stably transfected with small hairpin RNA (shRNA) directed against *GSTP1* or control shRNA lentiviral vector (Santa Cruz Biotechnology). Forty-eight hours post-transfection, cells at 60–70% confluence were infected with virus-containing media supplemented with 4 μg/mL polybrene (EMD Millipore, Billerica, MA, USA) overnight. Selection was performed using 2 μg/mL puromycin (Sigma-Aldrich). Protein expression and knockdown were confirmed by Western blotting using anti-p53 and anti-GSTP1 antibodies.

### Tumor xenograft and in situ apoptosis assays

All animal study procedures were performed in accordance with protocols approved by the Institutional Animal Care and Use Committee. Six-week-old athymic male nude mice (nu/nu) were purchased from Central Lab Animal Inc. (Seoul, Korea). AMC-HN9 cells (5 × 10^6^) were injected subcutaneously into each flank. Tumor volume and body weight were measured every other day. Tumors were measured using a caliper, and volume was calculated as (length × width^2^)/2. Treatment began when the cell implants became palpable nodules (= day 0). Mice were randomized into four treatment groups: vehicle, PL, cisplatin, and PL plus cisplatin.

Mice were treated by intraperitoneal (i.p.) injection of 2.5 mg/kg PL once per day, or by i.p. injection of 5 mg/kg cisplatin once per week, or with a combination of PL and cisplatin according to the same schedules. The mice were sacrificed on day 21, and tumors were isolated and analyzed by immunoblotting and *in situ* terminal deoxynucleotidyl transferase-mediated dUTP nick-end labeling (TUNEL) assay (R&D Systems, Minneapolis, MN, USA). The number of apoptotic bodies was counted blindly in ten randomly selected high-power fields. From mice with PL treatment or control, whole blood samples were collected from the tail vein and analyzed using an automated hematology analyzer (Beckman Coulter, Brea, CA, USA). For histological evaluation, normal tissues from vital organs, e.g., the oral cavity, lung, liver, kidney, spleen and small/large intestines, were isolated, fixed in formalin, paraffin-embedded, sectioned, and stained by hematoxylin and eosin. The statistical significance between different treatment groups was assessed by two-tailed Mann-Whitney *U*-test or Student's *t*-test.

## SUPPLEMENTARY MATERIAL AND FIGURES



## Abbreviations

HNC: head and neck cancer
PL: piperlongumine
ROS: reactive oxygen species
JNK: c-Jun N-terminal kinase
GSTP1: glutathione S-transferase pi 1
NAC: N-acetyl-L-cysteine
GSH: glutathione
GSSG: glutathione disulfide
DCF-DA: 2′,7′-dichlorofluorescein diacetate
PARP: poly(ADP-ribose) polymerase
siRNA: short interfering RNA
shRNA: small hairpin RNA
TUNEL: terminal deoxynucleotidyl transferase-mediated dUTP nick-end labeling
