# Supplementary material for: Piperlongumine selectively kills cancer cells and increases cisplatin antitumor activity in head and neck cancer
Source: Oncotarget. 2014 Aug 27;5(19):9227–38. doi: 10.18632/oncotarget.2402 (PMC4253430; doi:10.18632/oncotarget.2402)
Supplement: Supplementary file 1 [file oncotarget-05-9227-s001.pdf]

Piperlongumine selectively kills cancer cells and increases cisplatin antitumor activity in head and neck cancer

Supplementary Material

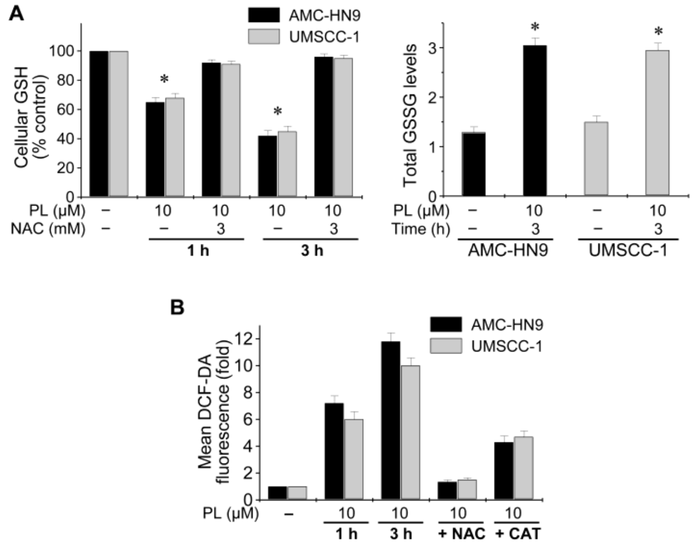

Supplementary Figure S1: Increased ROS accumulation by PL treatment in head and neck cancer cells. (*A*) Modulation of cellular GSH and GSSG levels by piperlongumine (PL). The GSH and GSSG levels were measured after AMC-HN9 (wild-type p53) and UMSCC-1 cells (p53-null) were exposed to PL for 1 h and 3 h with or without 3 mM NAC pretreatment for 1 h. The error bars represent s.d. from three independent experiments, each performed with triplicate samples. * denotes $p < 0.001$ relative to control. (*B*) ROS elevation by PL and prevention of the effect by NAC or catalase. AMC-HN9 or UMSCC-1 cells were exposed to 10 μM PL or DMSO (basal) for 1 h and 3 h. Cells were also pretreated with NAC (3 mM) for 1 h or catalase (CAT, 2,000 UmL^{-1}) for 2 h before exposure to PL (10 μM) for 3 h. ROS levels were measured by flow cytometry using DCF-DA and are shown as fold changes over DMSO-treated (basal) levels. Histograms are representative of three separate experiments. All values are the mean ± s.d. of three independent experiments.

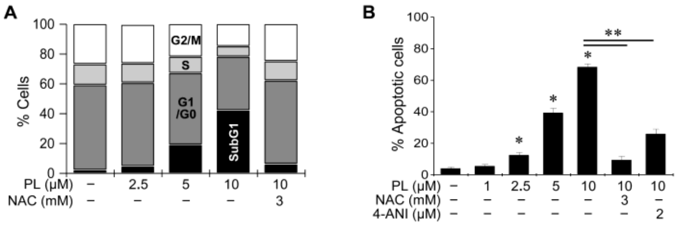

Supplementary Figure S2: Cell cycle changes and cell death induced by PL treatment in head and neck cancer cells. (***A***) Cell cycle analysis after exposure to PL. AMC-HN9 cells exposed to DMSO or PL for 48 h were stained with propidium iodide and subjected to flow cytometry analysis. (***B***) Apoptosis assays in AMC-HN9 cells exposed to PL. Cells were exposed to PL for 48 h, and the annexin V-positive apoptotic fractions were measured. *, ** denote $p < 0.01$ relative to control and 10 μM PL, respectively. Cells were also pretreated with 3 mM NAC for 1 h or 2 μM of the PARP inhibitor 4-amino-1,8-naphthalimide (4-ANI) for 16 h before being exposed to PL 10 μM.

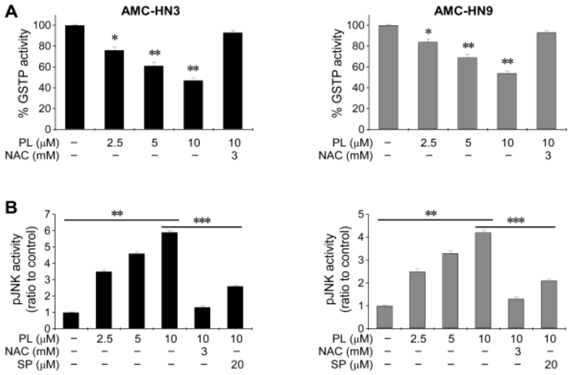

Supplementary Figure S3: Piperlongumine inhibits the enzymatic activity of glutathione *S*-transferase pi (GSTP) and activate JNK. (***A***) Inhibition of GSTP activity by PL treatment in head and neck cancer cells. (***B***) Activation of phospho-JNK (pJNK) by PL treatment. The enzymatic activity was measured using commercially available ELISA kits in AMC–HN3 and –HN9 cancer cells exposed to DMSO (control), PL, or combination of 10 μM PL and 3 mM NAC or 20 μM SP600125. The enzymatic activity of control was arbitrarily defined as 100% and the change or ratio in activity in PL-treated samples was plotted accordingly. The error bars represent the s. d. from three independent experiments, each performed in triplicate. *, ** denote $p < 0.05$ and $p < 0.01$ relative to the DMSO control, respectively. *** denote $p < 0.01$ relative to 10 μM PL treatment.

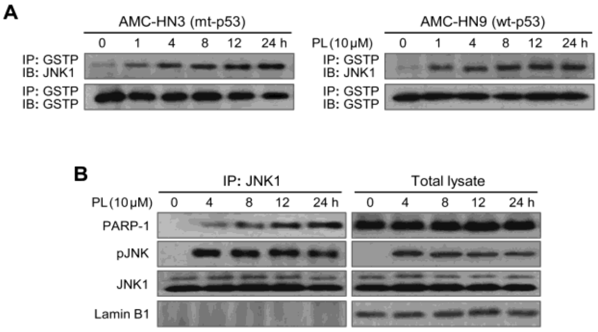

Supplementary Figure S4: Determination of protein-protein interaction between JNK and GSTP or PARP-1 after PL treatment. (***A***) Co-immunoprecipitation of GSTP1 with JNK. Protein-protein interactions between GSTP1 and JNK were evaluated by co-immunoprecipitation assays (IP) using the anti-GSTP1 antibody followed by immunoblotting (IB) with the antibody to JNK after exposing mutant p53 (mt-p53) AMC-HN3 cells and wild-type p53 (wt-p53) AMC-HN9 cells to 10 µM PL. (***B***) Co-immunoprecipitation between PARP-1 and JNK-1 upon PL treatment. AMC-HN3 cells were treated with 10 µM PL.

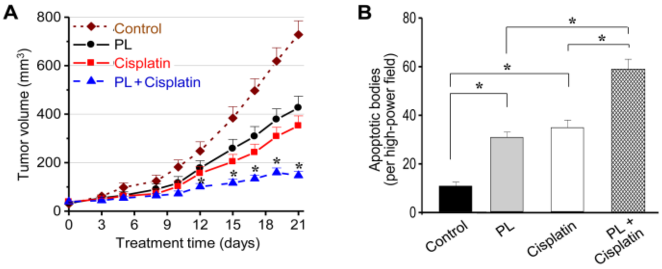

Supplementary Figure S5: Synergistic inhibition of *in vivo* tumor growth by PL and cisplatin in head and neck cancer cells. (***A***) Anti-tumor effect of PL and cisplatin in a tumor xenograft model. Nude mice were injected with 5×10^6 AMC-HN3 (mt-p53) cells in both flanks. Treatments with vehicle, PL, cisplatin, or the combination of PL and cisplatin began once the implanted tumor cells formed palpable nodules. Each group included eight mice. The error bars represent standard errors. * denotes $p < 0.05$ after day 12 between groups treated with PL or cisplatin and its combination.(***B***) Quantification from *in situ* TUNEL assay in tumor sections from each group. TUNEL-positive apoptotic bodies were counted blindly in 10 randomly selected high-powered fields. The error bars represent standard errors. Two-tailed Student's *t*-test, * denotes $p < 0.01$.
